# Role of noninvasive ventilation in weaning from mechanical ventilation in patients of chronic obstructive pulmonary disease: An Indian experience

**DOI:** 10.4103/0972-5229.60173

**Published:** 2009

**Authors:** Shiva B. N. Prasad, Dhruva Chaudhry, Rajan Khanna

**Affiliations:** **From:** Department of Clinical Immunology, Sanjay Gandhi Post Graduate Institute of Medical Sciences, Lucknow, India; 1 **From:** Department of Pulmonary and Critical Care, Pt. B.D Sharma PGIMS, Rohtak, India; 2 **From:** Department of Pulmonary and Critical Care, Pt. B.D Sharma PGIMS, Rohtak, India

**Keywords:** Chronic obstructive pulmonary disease, mechanical ventilation, noninvasive ventilation, respiratory failure, weaning

## Abstract

**Background:**

Endotracheal intubation and mechanical ventilation (MV) are often needed in patients of chronic obstructive pulmonary disease (COPD) with acute hypercapnic respiratory failure. The rate of weaning failure is high and prolonged MV increases intubation associated complications.

**Objective:**

To evaluate the role of Noninvasive ventilation (NIV) in weaning patients of chronic obstructive pulmonary disease (COPD) from MV, after T piece trial failure.

**Design:**

A prospective, randomized, controlled study was conducted in a tertiary care centre. 30 patients of acute exacerbation of COPD with acute on chronic hypercapnic respiratory failure, who were mechanically ventilated, were included in the study A T-piece weaning trial was attempted once the patients achieved satisfactory clinical and biochemical parameters. After T-piece failure, defined as pH < 7.35, PaCO_2_ >50 mmHg, PaO_2_ <50 mmHg, HR >100/min, RR >35, patients were randomized to receive either NIV or PSV.

**Results:**

Demography, severity of disease and clinical profiles were similar in both groups. No significant difference between the two groups in duration of MV (6.20 ± 5.20 days vs. 7.47 ± 6.38 days, *P* > 0.05), duration of weaning (35.17 ± 16.98 and 47.05 ± 20.98 hours, *P* > 0.05) or duration of ICU stay (8.47 ± 4.79 and 10.80 ± 5.28 days, *P* > 0.05) in Gp I and Gp II, respectively. Five patients developed VAP in the PSV group, where as only one patient had pneumonia in the NIV group. Lesser number of deaths in the NIV group at discharge from ICU (3 vs. 5 patients, respectively) and at 30 days (5 vs. 9 patients, respectively), it did not achieve statistical significance (*P* > 0.05).

**Conclusion:**

NIV is as useful as PSV in weaning and can be better in weaning failure especially in COPD for earlier weaning, decrease ICU stay, complications and mortality.

## Introduction

Removal of patients from mechanical ventilation (MV) has been termed liberation, discontinuation, withdrawal and most commonly weaning. The process of permanent removal of the artificial airway is extubation.[1] A balance must be achieved between the risk associated with early discontinuation and delay in extubation. Premature withdrawal causes loss of airway protection, cardiovascular stress, suboptimal gas exchange, muscle overload and fatigue. Delayed withdrawal exposes to complications associated with ventilation like infections, barotrauma, stretch injury, sedation, airway trauma and costs.[2]

Weaning strategies clearly affect the outcome and weaning should be aggressive but monitored carefully to minimise the risks. Two large trials have been performed comparing T-piece, assist control ventilation (ACV), intermittent mandatory ventilation (IMV) and pressure support ventilation (PSV) as approaches for weaning.[3,4] PSV was superior in one trial and T-piece in the other. Consensus regarding the best mode of weaning from these studies is difficult to derive. Patients of COPD requiring MV frequently suffer from persistent weaning failure, so early extubation with NIV decreases the duration of MV, length of intensive care unit (ICU) stay, incidence of nosocomial pneumonia and improves survival when compared to conventional weaning.[5] Noninvasive ventilation (NIV) is one of the new strategies in the weaning of patients of chronic obstructive pulmonary disease (COPD) needing MV. There are few studies which have systematically evaluated the role of NIV in weaning of patients of COPD on MV. Studies by Nava and Hilbert supported the role of NIV in weaning and postextubation respiratory failure.[6,7] Girault and Ferrer also found NIV as a suitable method for systematic extubation and weaning technique in patients with acute on chronic respiratory failure and persistent weaning failure.[8,9]

The objective of this study is to evaluate the effectiveness of NIV as a weaning method in patients of COPD on MV, in comparison to PSV.

## Materials and Methods

A two group, parallel, prospective randomized controlled trial was carried out in a tertiary care centre. Patients who were admitted in the intensive care ward, with acute exacerbation of COPD and needing intubation were eligible for the study. Acute hypercapnic respiratory failure in COPD was defined as severe dyspnoea in the absence of objectively documented causes such as pneumonia, and with arterial blood gas analysis (ABGA) findings of:

- pH <7.33 (breathing at room air)
- PaO_2_ <50 mmHg.
- PaCO_2_ >50 mm Hg

To make a decision for intubation as objectively as possible, the following guidelines were followed.[10]

Major:

- Respiratory arrest
- Loss of consciousness
- Psychomotor agitation requiring sedation
- Hemodynamic instability with systolic BP <70 mmHg or >180mm Hg
- Heart rate (HR) <50 beats/min with loss of alertness

Minor:

- Respiratory rate (RR) >35/min
- pH <7.30
- PaO_2_ <50 mm Hg
- Presence of weak cough reflex with accumulation of secretions
- Worsening of the neurological state (encephalopathy score)

The presence of one major criterion was considered as an indication for immediate intubation. The presence of two minor criteria after the failure of one hour of medical treatment and NIV was an indication for intubation. Patients who had concomitant neurological disease (other than hypercapnic encephalopathy), cardiac arrest, cardiogenic pulmonary oedema, cardiogenic shock, acute myocardial infarction, gastrointestinal perforation/obstruction, metabolic coma, coagulopathy and postoperative respiratory failure were excluded from the study.

Intubation was done through the orotracheal route. All patients were initially ventilated with control / assist control mode, in a stepwise manner (considering their level of consciousness, sedation level and improvement in ABG. Muscle relaxants and sedation were used as required. Standard ventilator settings for COPD i.e., respiratory rate of 12/min., tidal volume 8–10 mL/kg, FiO_2_ to obtain a saturation of 90% with a PEEP of 5 cm H_2_O and an I: E ratio of 1:2.5-3.0 was initiated. T-piece weaning trial was given to the patients when they were judged to have reached satisfactory neurological status, clinical and biochemical parameters with a SaO_2_ of 88% or more for a FiO_2_ of 40% after a minimum of 24 hours of ventilation.

The two hours T-piece trial failure was considered when the patient had any of the following:

- PaO_2_ <50 mm for a FiO_2_ of 40%
- pH < 7.35
- RR > 35/min.
- HR >145 bpm
- Systolic BP >180 mmHg or <70 mmHg
- Significant arrhythmia
- Agitation, anxiety or diaphoresis

Patients with T-piece trial failure were randomized into two groups to receive either NIV (group I) or continued weaning with invasive pressure support ventilation (group II). The Kendall and Babington table was used to randomize patients. Immediately after T-piece failure, patients were put back on CMV/ACV mode until previous PaCO_2_ and pH values were reached, with a respiratory rate of 30 per minute or less and then considered for the respective modality of weaning intervention.

After randomization, Group l patients to be weaned with NIV were then extubated and switched on to noninvasive pressure support ventilation with a full face mask using a BiPAP ventilatory assist system (RESMED, Sullivan-VPAP ST-II). Patients received NIV continuously except during meals and for expectoration. A particular level of IPAP and EPAP support that achieved t satisfactory blood gases and a RR <25/min were used. Once that was achieved, the pressure support was decreased by 2 cm of H_2_O every 4 hours with a good tolerance, and with close monitoring for any change in oxygen saturation and respiratory rate. As soon as we could reduce the inspiratory positive airway pressure (IPAP) and expiratory positive airway pressure (EPAP) levels to 8 and 4 cm of H_2_O, respectively, with a satisfactory ABG of PH ≥7.35, SaO_2_ ≥90%, FiO_2_ ≤40% and RR<30, patients were allowed to breathe spontaneously on a venturi mask. Group II, patients received pressure support ventilation with a particular level of pressure support that achieved satisfactory blood gases and a RR <25/min. Once that was achieved, the pressure support was decreased by 2 cm of H_2_O every 4 hours with a good tolerance and with close monitoring for any change in oxygen saturation and respiratory rate. As soon as the pressure support and PEEP reached 10 and 5 cm of H_2_O, respectively, with a satisfactory ABGA of PH ≥7.35, SaO_2_ ≥90%, FiO_2_ ≤40% and RR<30, patients were extubated and allowed to breathe spontaneously receiving oxygen therapy via a venturi mask.

Success of weaning was assessed after 2 hours of spontaneous breathing on a venturi mask by SaO_2_, FiO_2_, pH, RR, hemodynamic status, dyspnoea and a good neurological status. Successful weaning was defined when the patient maintained SaO_2_ ≥90%, FiO_2_ ≤40%, pH ≥7.35, respiratory rate <30/min with no dyspnoea and intact cognition. Absence of even one of these criteria was considered as weaning failure. Weaning was also considered a failure if the patient could not be taken off the ventilator after 30 days, or needed reintubation within 72 hours of disconnection from the ventilator, or if death related to MV occurred. Nosocomial infection, pneumothorax, ischemic event or fatal arrhythmias during the weaning process were considered causes of death associated with MV.

Arterial blood gas analysis (ABGA) was done at presentation and at 1, 4, 8 and 12 hours following the start of MV and also during the weaning process. Neurological score and APACHE II score were calculated at presentation. Ventilator associated pneumonia (VAP) was defined as the presence of new and persistent (>48 hours) lung infiltrates on chest radiography combined with fever, total leukocyte count (TLC) >10,000/μl after 48 hours on ventilator. Pulmonary function tests were performed with a portable spirometer as soon as the patients' clinical condition allowed testing before discharge from the intensive care unit. The predicted values for the local population were calculated by the previously published regression formula.[11]

The outcomes of treatment between the two groups were compared with the following parameters:

- Duration of MV, i.e. from the day of intubation to the day of extubation from the artificial airway (in group I before randomization and in group II before randomization plus weaning duration after randomization).
- Duration of ICU stay (from the day of admission to the day of discharge from ICU)
- Duration of weaning (after randomization).
- Incidence of nosocomial pneumonia
- Mortality at discharge from ICU and at 30 days discharge

Institutional review board approval was taken. Statistical analysis was done by using SPSS version 10.0

Results are given as mean ± SD. Chi-square test was applied for discrete variables and Mann-whitney U test for continuous variables where ever applicable. We also used the Cochrane Q test and Fisher exact test. Fisher exact test was used because sample size and values were small. Cochran's and Mantel-Hanazel test was used for test of independence between dichotomous variable and dichotomous response.

## Results

**Figure 1:**
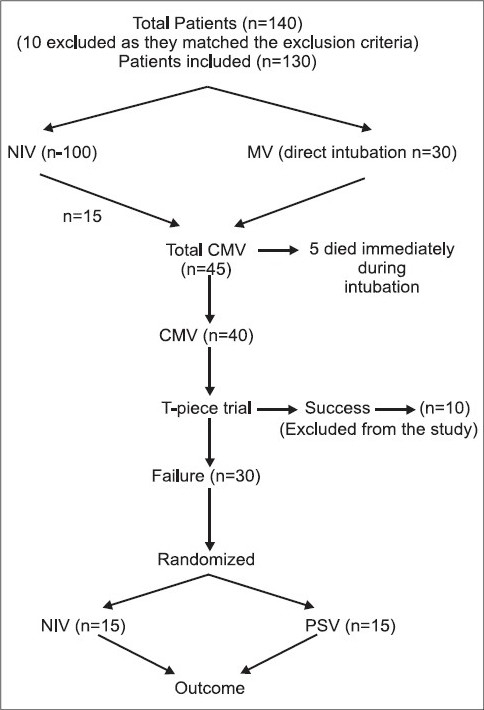
Methodology of the study

A total of 140 patients of acute exacerbation of COPD with acute on chronic type II respiratory failure were admitted in the intensive care unit (ICU) over a period of 18 months. Ten patients were excluded from the study, due to pneumonia (n =5), ischemic heart disease (n = 2), cardio-respiratory arrest (n = 2) and postoperative respiratory failure (n = 1). 100 patients were initially treated with NIV, of which 15 patients needed MV after one hour trial on NIV. 30 patients were taken on MV directly. A total of 45 patients needed MV and were eligible for the study. 5 patients died immediately after intubation. 10 patients were extubated directly after successful T-piece trial. The remaining 30 patients were randomized into 2 groups of 15 each [Figure 1].

The two groups were similar in terms of age, sex, smoking history, previous treatment, precipitating factors, severity of the disease, clinical and biochemical parameters during admission and at the time of randomization [Tables 1 and 2]. All patients were on irregular treatment with theophyllines, beta agonists and anticholinergics.

**Table 1:** Demographic profile

	Group I	Group II
Age (in years)	57.73 ± 11.23	61.13 ± 8.18*
Sex		
Male	12 (80%)	9 (60%)*
Female	3 (20%)	6 (40%)*
Smoking (Pack Years)	46.43 ± 17.03	35.45 ± 11.93*
Glasgow Coma Scale	8.40 ± 1.12	8.67 ± 1.45*
Heart Rate (per min)	133.47 ± 13.97	125.0 ± 19.90*
Respiratory Rate (per min)	33.80 ± 16.75	32.27 ± 15.92*
Mean Arterial Pressure (mm Hg)	72.93 ± 18.40	65.80 ± 18.44*
Total leukocyte count (per μl)	12,466.67 ± 4870.36	11,273.33 ± 3732.19*
PH	7.13 ± 0.06	7.13 ± 0.07*
PaCO_2_	95.98 ± 21.28	102.54 ± 28.36*

* *P* > 0.05

**Table 2:** Severity of disease

	Group I	Group II
FEV1 %	29.77 ± 6.98	29.33 ± 5.61*
APACHE II	30.73 ± 5.35	29.47 ± 5.00*

* *P* > 0.05

The mean IPAP and EPAP (inspiratory/expiratory positive airway pressure) initially used were 15.07 ± 1.27 and 6.21 ± 0.43 cm of H_2_O, respectively, in group I. The mean initial PSV used was 18.21 ± 1.1cm of H_2_O above PEEP (5 cm of H_2_O) in group II. There was no difference in the time spent on mechanical ventilation between the two groups i.e. 6.20 ± 5.20 day's *vs.* 7.47 ± 6.38 days in Gp I and Gp II, respectively (*P* > 0.05, Table 3). No difference in duration of weaning was observed between NIV and PSV groups (35.17 ± 16.98 and 47.05 ± 20.98 hrs, respectively, p.>0.05, Table 3). Patients weaned by NIV spent nearly 2 days less in the ICU, in comparison to weaning by PSV (8.47 ± 4.79 *vs.* 10.80 ± 5.28 days), the difference was not statistically significant (*P* > 0.05, Table 3). Mortality at discharge from ICU, three (20% mortality) and five deaths (33.33% mortality), respectively, were observed in the NIV and PSV group at the time of ICU discharge. Nevertheless, the difference was not statistically significant (*P* > 0.05, Table 3). At 30 days, there were lesser number of absolute deaths in the NIV group (5 *vs.* 9); however, it did not achieve statistical significance (*P* > 0.05, Table 3)

**Table 3:** Results

	Group I	Group II
Duration of ventilation (in days)	6.20 ± 5.20	7.47 ± 6.38*
Duration of weaning (in hours)	35.17 ± 16.98	47.05 ± 20.98*
Duration of ICU stay (in days)	8.47 ± 4.79	10.80 ± 5.28*
Death in ICU	3 (20%)	5 (33.33%)*
Death at 30 days	5 (33.33%)	9 (60%)*
Nosocomial pneumonia	1(6.66%)	5 (33.33%*)

* *P* > 0.05

Five patients (33.33%) in the PSV group suffered from VAP where as only one (6.66%) in the NIV group developed pneumonia. Other minor complications noted in the NIV group were claustrophobia (2 patients), skin abrasions (2 patients) and gastric distension (1 patient) [Table 4].

**Table 4:** Complications of NIV

Complications	Number of patients
Claustrophobia	2
Skin abrasion	2
Gastric distension	1
Pneumonia	1

## Discussion

NIV is recommended strongly in patients of chronic obstructive pulmonary disease with hypercapnic respiratory failure.[12] The requirement of prolonged MV in patients of COPD is due to impaired pulmonary mechanics, increased intrinsic PEEP, increased airway resistance with reduced pressure generating capacity of the muscles and pulmonary hyperinflation.[13,14] Whereas muscle fatigue and altered gas exchange are primarily responsible for weaning failure.[15] NIV allows the respiratory muscles to rest, and improves the patients' breathing pattern and gas exchange. Therefore, the patients who are likely to benefit from NIV are those with hypercapnic respiratory failure, a frequent situation in COPD and weaning failure.[16,17] NIV improves hypoxemia, hypercapnia and prevents rapid shallow breathing.[18]

There is less evidence supporting the efficacy of NIV in weaning. Few case reports and nonrandomized studies reported the beneficial effect of NIV in difficult to wean patients and increasing survival in patients needing prolonged ventilation.[19–21] Recent randomized control studies have shown that NIV decreases the duration of ventilatory support, length of ICU stay, incidence of Nosocomial pneumonia and improves survival when compared to conventional weaning techniques.[6–9] In the present study, we did not observe any significant statistical difference between the two groups regarding the duration of ventilation, weaning hours, ICU stay, VAP and mortality. In spite of small sample size in this study, there were two and four more deaths at discharge and 30 days follow up, respectively, in the group weaned by invasive PSV. The incidence of VAP was also 5 times higher (33.33% vs.6.66%) in the group weaned by PSV. Statistical significance was not achieved because study was under powered.

Increase in the duration of MV often leads to increased incidence of nosocomial pneumonia.[22–25] The presence of endotracheal tube predisposes to the development of nosocomial pneumonia by impaired cough reflex and mucociliary clearance. NIV has been one of the strategies to decrease the incidence of VAP.[26] Present study also confirms the same, as the incidence of VAP was 5 times less in the NIV arm of the study. NIV also decreases the mortality in the post extubation phase by avoiding sedation, tracheostomy, and intubation and allows swallowing, thereby decreasing gastro-oesophageal reflex, aspiration to the airways and VAP.[27–30]

The overall mortality in the present study is higher than the published data.[6,8] Nava *et al.* did not have mortality of any patient at the end of 60 days in the group weaned by NIV. In the study by Girault, the mortality was comparable between the two groups, weaned by PSV and NIV, respectively (2 *vs.* 0).[8] The higher mortality in our study relates to the severity of underlying disease, as evidenced by higher APACHE scores (≈30), low FEV1(<30%), severe acidosis and lower mean arterial pressures. VAP also contributed significantly to the mortality as all the patients having VAP died. Therefore, the patients included in the present study were not only sick but also had the severest form of COPD.

Nava concluded that NIV is more effective than PSV in weaning the patients of COPD from MV.[6] Whereas, Girault acknowledged it as a new and useful systematic approach to weaning.[8] Cochrane review has also demonstrated consistent positive effect on overall mortality in patients of COPD primarily by facilitating early weaning and extubation.[31] It concluded that NIV has a promising net clinical benefit. VENISE trial also supports the role of NIV in the management of difficult to wean patients of acute on chronic respiratory failure.[32] NIV not only reduces the need for ventilation and re-intubations, but also increases the survival.[6,10] The reason for the success of NIV is that it avoids complications of artificial airways. Esteban *et al.* also demonstrated the utility of NIV in preventing re-intubations in patients of COPD.[33] In the present study, NIV was as effective as PSV in weaning. It did reduce the complications associated with invasive ventilation and weaning like VAP and death in absolute terms. Present study however had one limitation, i.e. the numbers of patients studied were small.

In conclusion, NIV is as effective as PSV in weaning the patients of COPD with acute exacerbations from MV with some minor complications. Therefore, it should be the preferred weaning strategy for patients of COPD on MV in Indian scenario and otherwise. We believe that it is the first of the kind where there is direct comparison of PSV and NIV in weaning failed patients of COPD from India subcontinent.
